# Conceptual Models of Nursing in Critical Care

**DOI:** 10.1155/2021/5583319

**Published:** 2021-03-12

**Authors:** João V. Vieira, Sérgio Deodato, Felismina Mendes

**Affiliations:** ^1^Centre for Interdisciplinary Health Research, Universidade Católica Portuguesa, Lisbon 1649-023, Portugal; ^2^Department of Health, Instituto Politécnico de Beja, Beja 7800-111, Portugal; ^3^Comprehensive Health Research Centre, Universidade de Évora, Évora 7000-811, Portugal

## Abstract

**Introduction:**

Intensive care units are systems organized for the provision of care to patients in critical situations. In general, it is suggested that intensive care consists of a multidisciplinary and interprofessional specialty. Nevertheless, the predominance, relative to the professions that incorporate these units, falls on nurses. A conceptual model of nursing provides a framework for reflection, observation, and interpretation of phenomena and, specifically, it provides guidelines and guidance for aspects of clinical practice.

**Objective:**

To understand the applicability of conceptual models of nursing in intensive care units.

**Method:**

Review of the literature following the Scoping Review protocol of the Joanna Briggs Institute. The research was performed in CINAHL, Cochrane, Pubmed, Scopus, and Web of Science to identify studies published prior to 2021. Fourteen studies were selected.

**Results:**

There is no conceptual model of nursing universally accepted as ideal for intensive care units. However, there is unanimity in the identification of several benefits associated with the application of a conceptual model of nursing in the care of critically ill patients.

**Conclusion:**

For the selection of a conceptual model of nursing for these contexts, the focus should be on the person and the choice should fall on the model that is most appropriate to the patient, and not on the philosophy that supports the model. Considering the nature of care, the nursing team can select a model or a combination of models.

## 1. Introduction

Intensive care units are highly organized systems that aim to provide care to patients in critical situations. In these units, specialized nursing and medical care is provided, which includes a wide monitoring capacity and multiple modalities of physiological support of organs intended to preserve life during periods of insufficient organ systems that threaten it [1]. The high availability of resources existing in these units, namely, human, technological, and therapeutic, in addition to systematic advances in intensive care, gives these contexts a high capacity for monitoring and physiological support of the organ, which enables the care of people with highly critical and complex situations [2].

In general, it is suggested that intensive care consists of a multidisciplinary and interprofessional specialty, dedicated to the care of people who have developed or are at risk of developing acute organic dysfunction that puts them in a life-threatening situation. This specialty is based on technology that provides support for the necessary care in the event of failure of body systems [1]. The predominance, relative to the professions that incorporate these contexts, falls on nurses who are certainly the most representative professional class.

For contemporary knowledge of nursing, Fawcett [3] proposes a structural hierarchy that includes five components, from the most abstract to the most concrete, metaparadigm, philosophy, conceptual models, theories, and empirical indicators. This author argues that these components provide unique knowledge for the discipline and contribute to the distinction of nursing from other disciplines [3]. However, Meleis [4] argues that the use of multiple terminologies to define the concept of nursing theory, which is verified by the multiple variations of conceptions found in the literature, such as conceptual structure, conceptual model, paradigm, metaparadigm, theorem, and perspective, has been confusing.

The conceptual model is the third component of the structural hierarchy of contemporary nursing knowledge proposed by Fawcett [3]. This component consists of a set of relatively abstract concepts that address the phenomenon of central interest in a discipline. Although conceptual models have existed in the discipline of nursing since Florence Nightingale presented her ideas about nursing, the first conceptualizations about the discipline were not formally presented in the form of models [3].

A conceptual model of nursing provides a particular and distinct frame of reference through which people, their environment, and their health are perceived [5]. Its main function is to provide a framework for reflection, observation, and interpretation of phenomena and, specifically, it provides guidelines and guidance for aspects of clinical practice [5].

Several conceptual models have been formulated by nurses in an attempt to present a definition of what nursing is and what the nursing process implies [6] and, in general, these models present four central concepts for nursing practice, particularly, person, environment, health, and nursing [3]. The globality of conceptual nursing models can be adapted to different specialties of care practice. However, there are several factors to be considered when selecting a conceptual model to use in clinical practice [7].

In the particular case of intensive care units, similarly to other contexts, it is recognized that the use of conceptual nursing models makes it possible to differentiate clinical nursing practice from other health professions [7]. Apart from this, the implementation of a nursing model in these environments has potential benefits to patient care and has both educational and research value [8].

Fawcett et al. [7] state that several conceptual models can be implemented in intensive care, even though they do not recommend some models for certain patients.

In view of the considerations previously presented about the conceptual models of nursing and their implementation in intensive care units, with this review the authors intend to identify which conceptual models of nursing are possible to implement in intensive care units.

## 2. Methods

This is a literature review adhering to the Scoping Review protocol of the Joanna Briggs Institute [9], including the development of the research question, research in scientific databases, identification of inclusion and exclusion criteria, selection of studies, analysis and interpretation of the selected studies, and synthesis and presentation of results.

To formulate the research question, the PCC mnemonic was used: (P) Population, (C) Concept, and (C) Context. The following question was asked to answer the outlined objective that served as the guiding principle for this literature review: what conceptual models of nursing (Concept) can be implemented in the process of caring for the critically ill patient (Population) admitted to the intensive care unit (Context)?

The research strategy included a search for studies published in French, English, Spanish, and Portuguese carried out by the three authors, independently, to identify studies published until 2021 in CINAHL Complete, Cochrane, Pubmed, Scopus, and Web of Science databases. The search included the descriptors nursing model, nursing conceptual model, nursing theory, intensive care unit, intensive care units, ICU, critical care, ICU, and critically ill. The descriptors were connected with the Boolean operators “AND” and “OR” in the following arrangement: “nursing model” OR “nursing conceptual model” OR “nursing theory” AND “intensive care unit” OR “intensive care units” OR “critical care” OR “ICU” OR “critically ill”.

This review included exclusively studies on conceptual nursing models and/or nursing theory applied to intensive care units. Studies with methodologies that focus on the object of study, from academic journals, with analysis by specialists and with available references, were privileged. All studies with ambiguous methodology, with no correlation with the object of the study, or presenting conflicts of interest were excluded.

The initial search identified 616 results, specifically, CINAHL (*n* = 196), Cochrane Central Register of Controlled Trials (*n* = 8), Cochrane Database of Systematic Reviews (*n* = 0), PUBMED (*n* = 181), Scopus (*n* = 194), and Web of Science (*n* = 37), from other sources (*n* = 0), ordered by relevance or best match, with 252 duplications identified.

**Figure 1:**
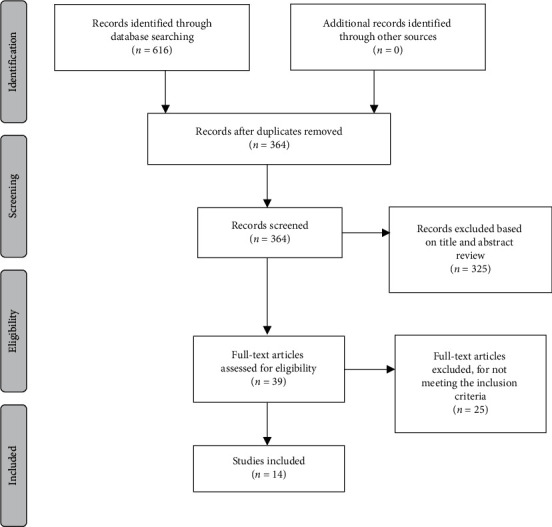
Literature search process—preferred items presented in the Joanna Briggs Institute Guidance [9].

The evaluation of the remaining 364 results, carried out by three authors independently, proceeded in two phases, namely, the phase of selecting the studies to be analyzed after reading the titles and the abstract, which allowed the identification of 39 studies with potential interest for the review; the phase of full reading of all studies, after which, once the inclusion criteria and analysis of the levels of evidence and methodological quality were applied, 14 studies were selected (Figure 1).

## 3. Results

It was decided to present the results obtained by analyzing the studies included in this review in table format (Table 1) to facilitate and simplify their reading and interpretation. The results are presented chronologically.

## 4. Discussion

The authors of the studies selected for this review analyzed several conceptual models of nursing, particularly their implementation in intensive care and application in the care of critically ill patients.

In the study by Fawcett et al., three conceptual nursing models and their implementation in intensive care were evaluated, Levine's Conservation Model, Neuman's System Model, and Dorothea Orem's Self Care Model [5]. The authors state that, regardless of the model selected to guide the provision of nursing care, the application of a conceptual model of nursing in these environments provides holistic care to people who experience life-threatening situations and contributes to the improving the quality of care [5].

Johnson presents a study in which it is concluded that no conceptual model had reached universal acceptance for implementation in intensive care [10]. The author states that, for application in the practice of care, especially in intensive care, the concepts of a conceptual model must be possible to reduce to empirical terms and must be possible to use in conjunction with the medical model and, frequently, overlap that model [10].

McClune and Franklin present a study in which they evaluate the mead model for nursing and its implementation in intensive care, a model that arises from the adaptation of the Roper, Logan, and Tierney Model [11]. According to the authors, the Roper, Logan, and Tierney Model proved to be an appropriate choice for these environments. Nevertheless, some problems were identified in terms of care planning, mainly related to nursing interventions related to life activities, and the terminology of the nursing process. In the mead model, the person is the centre of the model, instead of the activities of life. It should also be noted that this model attaches great importance to the factors that influence the person and their health, an aspect that is of fundamental importance in intensive care [11]. Sutcliffe also evaluated the applicability of the mead model in intensive care and concluded that this model, although not perfect, can be very useful to guide nursing practice, since it supports nursing knowledge and its objectives and beliefs, in addition to being practical and “user friendly” [15].

In the study of Sommers, five participants present their opinions regarding the implementation of conceptual models of nursing in intensive care [12]. Most agree that in intensive care nursing practice there are benefits from combining several conceptual models, the most mentioned of which include: Roy's Adaptation Model; Levine's Conservation Model; Neuman's Model; Roper, Logan, and Tierney's Model; Humanistic Nursing Theory of Paterson and Zderad; Orem's Model [12].

In the study by Jacobs, Orem's conceptual model is evaluated [13]. The author concludes that, despite the unquestionable benefits of this model, the possibilities of achieving self-care in intensive care units are very limited, especially due to society's global perception of critical illness, which attributes the responsibility of recovery to the health team. However, despite this limitation, it is proven that the implementation of this model contributes to the promotion of personalized care and enhances the importance of the perception of the person as a whole [13].

Boström at al. carried out a study in which they sought to assess the quality of care in intensive care units, using Henderson's conceptual model for the elaboration of the observation and question scheme [14]. The authors found significant improvements in interventions aimed at assisting in basic physical activities, particularly breathing, nutrition, elimination, mobilization, sleeping and resting, and avoiding dangers, but did not identify any benefit in interventions aimed at assisting psychological or spiritual needs [14].

In the study by Fawcett et al., it is proposed that, for the selection of a conceptual model of nursing for intensive care, the focus should be more directed to the person, instead of being directed to the philosophy of nursing itself; that is, more than forcing the patient to fit into a particular conceptual model, the selection of the conceptual model should be for the one that is most appropriate for the patient [7]. The authors also propose that, for the model selection, the following questions should be answered: Does the model cover all the declared problems or needs of the patient? Is the nursing objective proposed by the model consistent with the objectives related to the patient's health? Are the nursing interventions associated with the nursing model consistent with the patient's expectations for nursing care [7]?

In the first study of the 2000s that was included in this review, Chan considers that the Roy Adaptation Model can provide a comprehensive systematic guide based on what nurses most often identify in terms of physiological function and that it can promote a clear identification of the patient problems in terms of physiological function and, with that, prioritize care more efficiently [16]. According to this author, the incorporation of a conceptual model of nursing as a framework to guide nursing practice in an intensive care unit enables nurses to understand patient's needs and offers them a clear definition for delivering patient care [16].

Kaplow and Reed present the first study that was identified for inclusion in this review in which the AACN Synergy Model is evaluated, a model that describes nursing practice based on eight critical patient characteristics and eight nurse competencies [17]. According to these authors, the core of this model is that the needs and characteristics of patients and families influence and drive the characteristics and competencies of nurses and they suggest that this model is an excellent framework for the organization of care for critically ill patients [17].

The study provided by Truppel at al. proposes that the conceptual model of Wanda Horta and its respective nursing process in the assistance practice in an intensive care unit support clinical judgement, support the planning of interventions to implement with scientific evidence, and contribute to the definition of nurses' performance [18]. Perão et al., the authors of the most recent study included in this review, also evaluated the implementation of Wanda Horta's theory in intensive care and concluded that the appropriate implementation of this theory in these environments can contribute to the safety of care provided and that the relationship between nursing theory and care security reinforces the scientific nature of the discipline [20].

The last two studies included in this review do not propose a specific nursing conceptual model for implementation in intensive care. According to Robb, conceptual models have already proved their importance and have earned a place in the practice of intensive care, and more research is needed to prove their effectiveness in improving care to critically ill patients [8]. The selection of the conceptual nursing model for these environments will always be influenced by the nature of the care provided by the multidisciplinary team [8]. Reinoso and Núñez acknowledge that the implementation of a conceptual model of nursing in intensive care contributes to the incorporation of knowledge of the discipline of nursing in clinical practice, in addition to promoting the increase of awareness of professional and quality care [19]. The authors add that the continuous monitoring and evaluation of the implemented model are essential and allow for necessary adjustments, changes, and modifications [19].

## 5. Conclusions

There is no consensus on the implementation of a specific conceptual model of nursing in intensive care units. Some authors propose some models and identify several benefits of their implementation in intensive care, which include contribution to the provision of holistic care to people who experience life-threatening situations, promoting quality of care, improving care security, enabling an individualized approach and promoting personalized care, prioritizing the identification of nursing problems and interventions to be implemented, contributing to the organization of care, promoting the incorporation of scientific knowledge in clinical practice.

The authors of this review highlight the fact that, for the selection of a conceptual model for these contexts, the focus should be on the person and not on the philosophy that supports the model; that is, the choice must fall on the model that is most appropriate to the patient. This includes the possibility of combining different models and must be made by the nursing team according to the nature of the care provided. The conceptual model of Virginia Henderson and the mead model, an adaptation of the Roper, Logan, and Tierney Model, seem to be suitable options for implementation in intensive care, in combination or in isolation. Virginia Henderson's model contributes to significant improvements in basic physical activities. The mead model suggests that the person is the centre of care and attaches enormous importance to the factors that influence the person and their health, besides being a model that can be extremely useful to guide nursing practice, since it supports nursing knowledge, and it is practical. Nevertheless, there are several conceptual models of nursing in addition to those that were analyzed by the authors of the studies included in this review, so it cannot be excluded that others can be successfully implemented in intensive care units.

With the realization of this study, the authors confirm that few studies were carried out on the implementation of conceptual models of nursing in intensive care units, especially in the last two decades. The authors are unanimous in considering that more studies should be carried out, especially primary studies, with more significant samples.

## Figures and Tables

**Table 1:** Synopsis of the analyzed studies.

Authors (year)	Title	Author's conclusions
Fawcett et al. [5]	Conceptual models of nursing: application to critical care nursing practice	Regardless of the choice, the use of a conceptual model in intensive care provides holistic care to people who experience life-threatening situations and contributes to the quality of care provided.
Johnson [10]	Evaluating conceptual models for use in critical care nursing practice	The concepts of a conceptual model must be reducible to empirical terms and must be possible to use in conjunction with the medical model. No conceptual model has achieved global acceptance for implementation in intensive care.
McClune and Franklin [11]	The mead model for nursing—adapted from the roper/Logan/Tierney model for nursing	With the mead model, the person is the centre of the model and importance has been attached to the factors that influence the person and health, due to the fundamental importance that they have in intensive care.
Sommers [12]	Interviews: opinions on conceptual models from other critical care nurses	Most participants agree with a combination of conceptual models in critical care practice.
Jacobs [13]	Orem's self-care model: is it relevant to patients in intensive care?	The application of Orem's self-care model promotes an individualized approach to nursing care and enhances the importance of perceiving the patient as a whole.
Boström et al. [14]	Nursing theory-based changes of work organization in an ICU effects on quality of care	With the application of Henderson's nursing model in an intensive care unit, improvements were identified in interventions that aimed to assist in basic physical activities. There has been no improvement in activities aimed at assisting psychological or spiritual needs.
Fawcett et al. [7]	Guidelines for selecting a conceptual model of nursing: focus on the individual patient	For the selection of a conceptual model of nursing for intensive care units, the focus should be more directed to the person, instead of being directed to the philosophy of nursing.
Sutcliffe [15]	Philosophy and models in critical care nursing	The mead model is useful to guide nursing practice, once it embodies nursing knowledge, goals, and beliefs, besides being practical and “user friendly.”
Robb [8]	Do nursing models have a place in intensive care units?	The selection of a nursing model in intensive care is influenced by the multidisciplinary nature of the area of care. Further research is needed to provide the effectiveness of conceptual models of nursing in improving patient care.
Chan [16]	Using the Roy adaptation model to guide the health assessment of patients in an intensive care setting in Hong Kong	Roy adaptation model promotes a clear identification of the patient problems and prioritizes care more efficiently and can provide a comprehensive systematic guide based on what nurses identify in terms of physiological function.
Kaplow and Reed [17]	The AACN synergy model for patient care : a nursing model as a force of magnetism	The AACN synergy model is an excellent framework to organize the work of patient care throughout the health care system.
Truppel et al. [18]	Nursing assistance practice at an intensive therapy unit supported by the conceptual model of Horta	The conceptual model of Horta and its respective nursing process support the clinical judgement and the planning of the interventions to be implemented with scientific evidence and help with the definition of the nurse's performance.
Reinoso and Núñez [19]	Nursing models in critical care units: step toward advanced nursing care	The implementation of a model of nursing in intensive care contributes to the incorporation of knowledge of the discipline in clinical practice.
Perão et al. [20]	Patient safety in an intensive care unit according to Wanda Horta's theory	An appropriate implementation of Wanda Horta's theory in intensive care can contribute to the safety of care in these environments and it reinforces the scientific nature of nursing.
